# Cinematic rendering in the evaluation of LVAD outflow tract compression

**DOI:** 10.1093/ehjimp/qyag028

**Published:** 2026-02-13

**Authors:** Amy Avakian, Jay S Leb, Muhammad Umair

**Affiliations:** Southern Hills Hospital and Medical Center, 9300 W Sunset Road, Las Vegas, NV, USA; Department of Radiology, Columbia University, New York, NY, USA; Department of Radiology, Columbia University, New York, NY, USA

## Clinical presentation

A 62-year-old male with a HeartMate 3 left ventricular assist device (LVAD) implanted 18 months prior presented with recurrent low-flow alarms and progressive dyspnoea on exertion. Device interrogation revealed decreased power consumption and estimated flow. Transthoracic echocardiography (TTE) suggested inadequate left ventricular unloading but was limited by acoustic shadowing from the LVAD components, precluding definitive assessment of the outflow cannula. Given the clinical presentation and inconclusive echocardiographic findings, cardiac computed tomography angiography (CTA) was performed to evaluate for mechanical LVAD complications.^1–3^ CTA identified biodebris accumulation external to the outflow graft causing extrinsic compression of the inner cannula. Biodebris accumulation represents an emerging complication of HeartMate 3 devices, occurring in approximately one-third of patients with adequate imaging and correlating with longer duration of LVAD support.^4^ This material can accumulate either internal or external to the outflow graft, leading to progressive luminal narrowing as appreciated in *Video 1* and *Video 2*.^4,5^

## Imaging findings

**Figure 1:**
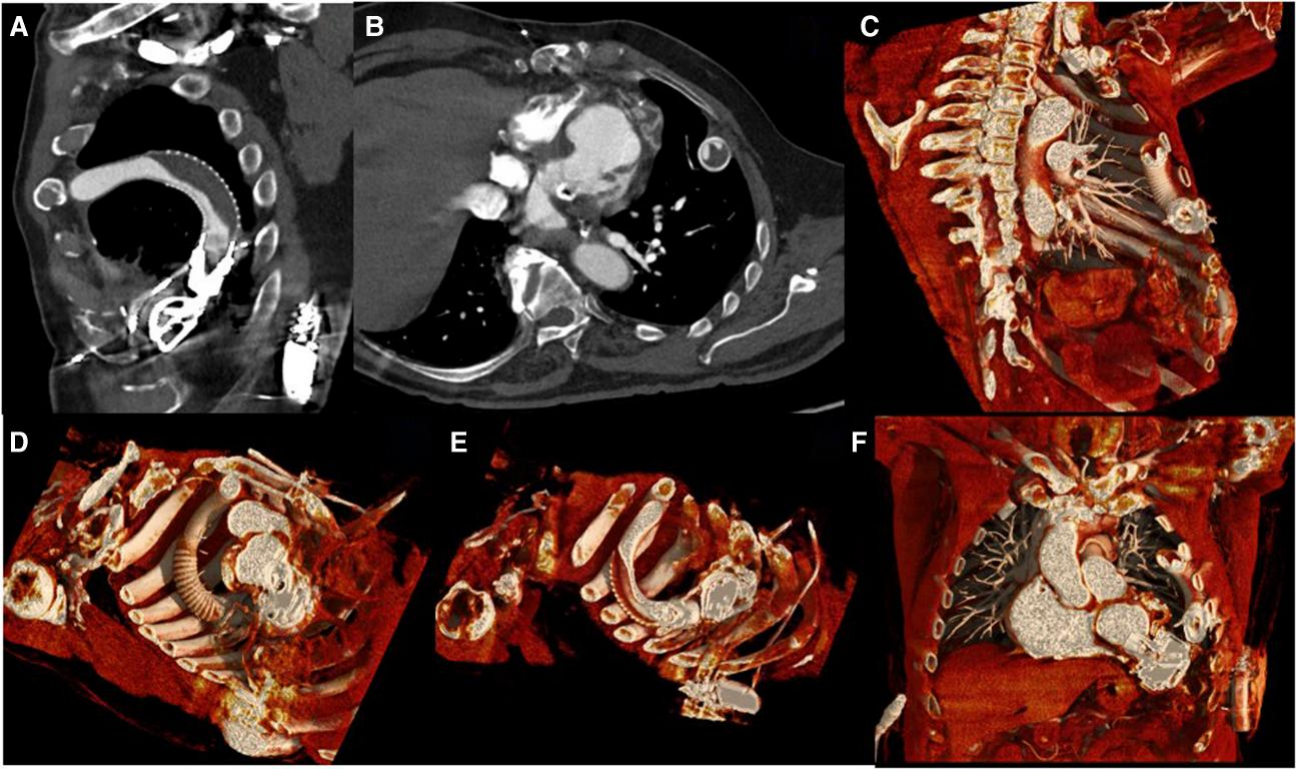
Computed tomography angiography and cinematic rendering of LVAD outflow tract compression from biodebris accumulation. (*A*) Two-dimensional oblique sagittal reformatted CTA image centred at the point of maximal compression in the LVAD outflow tract. The outflow cannula courses through the thorax with biodebris build-up (white arrow) causing external compression of the inner cannula. (*B*) Two-dimensional double orthogonal luminal view of the LVAD outflow graft in the region of biodebris accumulation. Crescent-shaped biodebris causes narrowing of the mid-portion of the inner cannula lumen (white arrow). (*C*) Three-dimensional cinematic rendering of the LVAD outflow cannula demonstrating the general course from the pump housing to the ascending aorta. The area of luminal compression in the mid-outflow graft is indicated (white arrow). The outflow graft anastomosis to the ascending aorta is marked (white arrowhead). Separate colour settings differentiate the biodebris, inner cannula lining, and blood pool. (*D*) Three-dimensional cinematic rendering with segmentation demonstrating the external features of the outflow cannula. The extent of biodebris accumulation along the external surface of the outflow graft is indicated (white arrow). The inflow cannula at the left ventricular apex is marked (arrowhead). (*E*) Cut-surface segmentation with the outflow cannula partially cut-open to demonstrate biodebris collection with associated extrinsic compression of the inner lumen (white arrow). This view clearly demonstrates that the obstruction results from external compression rather than intraluminal thrombus, as the biodebris is located between the outer and inner cannula walls. The invaginated appearance of the inner lining under pressure from biodebris build-up is evident. (*F*) Cinematic rendering assessment of the LVAD inflow cannula demonstrating device position and orientation. The inflow cannula tip is positioned within the left ventricular apex (white arrow) with the cannula oriented toward the interventricular septum, representing somewhat suboptimal positioning in the setting of myocardial thinning from prior infarcts. A portion of the outflow cannula is also visualized.

A CTA-based two-dimensional oblique sagittal reformatted image centred at point of action in the LVAD outflow tract demonstrating LVAD outflow cannula coursing through the thorax with bio-debris build-up leading to external compression of the inner cannula (*Figure 1A*). A CTA-based two-dimensional double orthogonal luminal view of the LVAD in the region of bio-debris build-up leading to about 70% narrowing of the inner cannula, however, comprehensive assessment of the whole outflow cannula will require multiple reformats at different angulations (*Figure 1B*).

Three-dimensional reconstruction of the LVAD outflow cannula demonstrates the general course of the LVAD outflow cannula and visualizes both the outer surface and lumen of the cannula with luminal compression noted (*Figure 1C*). Separate colour settings are applied for the bio-debris, the inner cannula lining, and the blood pool, clearly differentiating these structures. In addition to this level of detail, global illumination adds an additional layer of photorealistic appeal by providing real-life-like lighting and shadowing (*Figure 1C*). A key strength of this imaging approach is the ability to distinguish external compression from intraluminal thrombus by identifying the precise location of the biodebris external to the inner cannula wall, a critical distinction for surgical planning that is difficult to appreciate on echocardiography.^2,6^ Global illumination adds photorealistic lighting and shadowing, providing intuitive spatial relationships that may aid in pre-procedural planning.^7,8^

Cinematic rendering enables visualization of the entire outflow cannula within a single three-dimensional volume. Most of the outflow cannula is captured within a single imaging volume, segmented to demonstrate the external features of the outflow cannula (*Figure 1D*, white arrow indicating biodebris accumulation). Cut-surface segmentation in cinematic rendering applications can be applied in any desired plane to cut-open a vessel or luminal structure. The outflow cannula is partially cut-open to demonstrate biodebris collection with associated extrinsic compression of the inner lumen (*Figure 1E*). The invaginated appearance of the inner lining of the outflow cannula under pressure from biodebris build-up is clearly evident.

Cinematic rendering assessment of the LVAD inflow cannula demonstrates inflow cannula orientation without evidence of intraluminal thrombus, confirming inflow patency and visualization of the device position (*Figure 1F*). The inflow cannula tip is positioned within the left ventricular apex with the cannula oriented toward the interventricular septum, representing somewhat suboptimal positioning in the setting of myocardial thinning from prior infarcts. A portion of the outflow cannula is also visualized.

## Clinical significance

While standard two-dimensional CTA imaging can detect outflow graft obstruction, cinematic rendering provides comprehensive visualization of the entire outflow cannula within a single three-dimensional volume and offers intuitive spatial relationships through photorealistic lighting and shadowing.^7–9^ This case demonstrates how cinematic rendering complements standard imaging by clearly differentiating external compression from intraluminal thrombus, information critical for determining whether endovascular stenting or surgical revision is appropriate.^2,3^

Echocardiography remains the first-line imaging modality for LVAD complications due to its accessibility and ability to assess ventricular function, filling pressures, and response to speed adjustments.^10,11^ However, TTE has inherent limitations due to acoustic shadowing from LVAD components and cannot visualize the entire outflow graft.^10^ CTA provides complementary information by allowing direct, complete visualization of the LVAD system, including inflow cannula position and the entire course of the outflow graft.^6,12^ The combination of TTE and CTA yields superior diagnostic performance compared with either modality alone, with sensitivity of 67%, specificity of 93%, and diagnostic accuracy of 73% for identifying cardiomechanical LVAD complications.^6^

Following CTA diagnosis, the patient underwent successful endovascular stent placement of the outflow graft with resolution of low-flow alarms and symptomatic improvement. Patients with external compression from biodebris have been successfully treated with endovascular stenting, whereas intraluminal thrombosis typically requires surgical revision.^2,3^ One-year survival after identification of outflow graft narrowing is 93%, with most patients maintaining adequate left ventricular unloading.^4^

## Data Availability

No new data were generated or analyzed in support of this research.

## Lead author biography

Muhammad Umair, MD is an Assistant Professor of Radiology at the NewYork Presbyterian Hospital—Columbia University, with a strong focus on cardiovascular imaging (MRI and CT). His advanced training included a Diagnostic Radiology residency and an Early Specialization in Interventional Radiology (ESIR), followed by a dedicated Cardiovascular Imaging fellowship, all completed at Northwestern University—one of the nation’s premier centres for both interventional and cardiovascular imaging. Previously, Dr Umair practiced at the Johns Hopkins Hospital and served as an Assistant Professor of Radiology with the Johns Hopkins University School of Medicine. His primary research interests revolve around novel imaging modalities that can significantly enhance patient outcomes. These include 4D flow MRI, CMR parametric mapping (such as T1 and extracellular volume mapping), and the application of quantitative imaging biomarkers in the evaluation of cardiomyopathies (e.g. amyloidosis, diabetic cardiomyopathy, and arrhythmogenic right ventricular cardiomyopathy), as well as advanced 3D visualization techniques including cinematic rendering (CR). He also investigates cost-effectiveness analyses of advanced cardiac imaging—especially CT–Fractional Flow Reserve (CT-FFR) and CMR-based assessments—to ensure that the implementation of new imaging techniques remains equitable, efficient, and beneficial to patients within a value-based care framework. A key dimension of his work is the ethical and legal integration of artificial intelligence (AI), including generative AI platforms, into cardiovascular imaging. His goal is to develop transparent, bias-resistant, and patient-centred AI applications that elevate diagnostic accuracy while upholding data privacy and broad accessibility. In addition, he participates in global outreach initiatives aimed at improving cardiac MRI availability in low-resource settings. By collaborating with international teams, including the Consortium for Advancement of MRI Education and Research in Africa (CAMERA), he has helped deliver remote education, training, and technology solutions for radiologists and technologists working under resource constraints.
